# Prevalence and determinants of oral and cervicogenital HPV infection: Baseline analysis of the Michigan HPV and Oropharyngeal Cancer (MHOC) cohort study

**DOI:** 10.1371/journal.pone.0268104

**Published:** 2022-05-16

**Authors:** Andrew F. Brouwer, Lora P. Campredon, Heather M. Walline, Brittany M. Marinelli, Christine M. Goudsmit, Trey B. Thomas, Rachel L. Delinger, Yan Kwan Lau, Emily C. Andrus, Monica L. Yost, Jodi K. McCloskey, Taylor S. Sullivan, Alex S. Mortensen, Suiyuan Huang, Keith Murphy, Bonnie Cheng, Kayla Stanek, Thankam Nair, Thomas E. Carey, Rafael Meza, Marisa C. Eisenberg

**Affiliations:** 1 Department of Epidemiology, University of Michigan, Ann Arbor, Michigan, United States of America; 2 Department of Otolaryngology, University of Michigan, Ann Arbor, Michigan, United States of America; Ruđer Bošković Institute, CROATIA

## Abstract

We determined baseline oral and cervicogenital human papillomavirus (HPV) prevalence and determinants of infection in the Michigan HPV and Oropharyngeal Cancer (MHOC) study. We enrolled 394 college-age and older participants of both sexes in Ann Arbor, Michigan and the surrounding area. All participants provided an oral sample at baseline, and 130 females provided a cervicogenital sample. Samples were tested for 18 HPV genotypes using polymerase chain reaction (PCR) MassArray. Participants filled out sociodemographic and behavioral questionnaires. Prevalence ratios for HPV oral or cervicogenital prevalence by predictor variables were estimated in univariable log-binomial models. Analysis was conducted 2018–20. In the full cohort, baseline oral HPV prevalence was 10.0% for any detected genotype (among the 338 valid oral tests at baseline) and 6.5% for high-risk types, and cervicogenital prevalence was 20.0% and 10.8%, respectively (among the 130 first valid cervicogenital tests). Oral HPV prevalence did not vary by sex, with 10.5% of women and 9.0% of men having an infection. We found a high prevalence of oral and cervicogenital HPV infection in college-age participants reporting no lifetime sexual partners. Reporting a single recent partner was associated with a lower oral HPV prevalence (PR 0.39, 95% CI: 0.16, 0.96) than reporting no recent (but at least one ever) partner. No similar protective effect was seen for cervicogenital HPV. Both oral and cervicogenital prevalence increased with the number of recent partners for most sexual behaviors. We observed an ecological fallacy masking the direction of impact of vaccination on HPV prevalence in the full cohort compared to the college-aged and the age 23+ populations considered separately. Substance use was not significantly associated with oral or cervicogenital HPV infection. Many studies report substantially higher oral HPV infection prevalence in men than in women. That difference may not be uniform across populations in the US.

## Introduction

While the human papillomavirus (HPV) is well known as the primary cause of cervical cancer, incidence of HPV-related oropharyngeal squamous cell carcinoma (OPSCC) in the U.S. is now greater than that of cervical cancer [1–4]. Indeed, an increasing fraction of OPSCCs are attributable to HPV [5–8], with some studies reporting over 80% of OPSCC cancer patients testing positive for a high-risk HPV genotype, i.e., a genotype that can cause cancer [9]. Understanding the determinants, prevalence, and dynamics or oral HPV infection will be essential for designing interventions.

OPSCC is more than three times more common in men than in women in the U.S. [1, 4], which mirrors higher prevalence of oral HPV in men. Nationally representative studies in the U.S. have estimated prevalence of oral HPV as approximately 11% in men and 4% in women [10, 11]. Oral sex is a known risk factor—and likely primary transmission pathway—for oral HPV [12–15], and there may be risk differences based on the sex of one’s partner [16]. More broadly, sexual behaviors and smoking have been identified as important risk factors of oral HPV infection [17]. Nevertheless, questions remain about which risk factors are in fact causally related to transmission and which are correlated with transmission behaviors. Moreover, the impact that HPV vaccination will have on oral HPV prevalence [18, 19] and transmission dynamics [20] needs to be elucidated.

The Michigan HPV and Oropharyngeal Cancer (MHOC) Study aims to evaluate patterns of oral HPV infection, prevalence, incidence and clearance and their relationship to sexual history and sexual behaviors. MHOC includes an epidemiological arm that tests a longitudinal cohort for oral (and, in a subset of participants, cervicogenital) HPV [21]. The longitudinal HPV infection and sexual history data will facilitate the development of individual-based network models of HPV transmission and will be used to determine parameter values for multiscale models of HPV-related OPSC carcinogenesis. This first, hypothesis-generating analysis, we present the baseline prevalence of oral and cervicogenital HPV and the association of infection with demographic and behavioral characteristics in the MHOC Study.

## Methods

We previously published the full study protocol [21], including both the baseline and longitudinal aspects of the study. Here, we briefly describe the main aspects of the study related to the baseline prevalence examined here.

### Study subjects

Study participants were recruited in Ann Arbor, Michigan and the immediate surrounding areas. Participants were recruited at University of Michigan campus residence halls, through community fliers, and through the University of Michigan Health Research website. Volunteers over the age of 18 without a history of head and neck cancer who were willing to participate in both the baseline and longitudinal (3–4 visits per year for 3 years) portions of the study were invited to enroll. We enrolled participants between April 2015 and December 2017. A substudy focusing on cervicogenital HPV enrolled participants that had a vagina and were not pregnant to enroll; these participants could not collect cervicogenital specimens on days that they were menstruating. Documented informed consent was obtained from all participants. The University of Michigan Institutional Review Board approved consent documents and study protocol (HUM00090236).

### Surveys

A baseline questionnaire was administered to each participant at their initial visit. Participant ID numbers were assigned to ensure participant confidentiality. The surveys were designed to individually assess a variety of topics including demographics, vaccination and screening history, sexual health and behavior, and alcohol and drug use. Sexual behavior questions assessed current and past experiences of vaginal, oral, and anal sex, including the number of ever and recent (past 6 months) sexual partners with whom they engaged with each of these types of sex. Vaginal and anal sex were defined to participants as vaginal and anal penetration of any kind, respectively, and oral sex was defined as stimulation of genitals by the mouth.

### HPV testing

All participants self-collected a saliva sample with Scope mouthwash (Proctor & Gamble; Cincinnati, OH) as in NHANES [22] or an Oragene RE-100 kit (DNA Genotek; Kanata, Canada) [23]. Saliva samples were taken at each study visit. Participants who had a vagina, were not pregnant, and were not menstruating at the time of a study visit were invited to self-collect a cervicovaginal sample with a HerSwab (Eve Medical; Toronto, Canada). DNA was extracted from samples and genotyped using polymerase chain reaction (PCR) MassArray [9]. We tested for 18 genotypes: 6, 11, 16, 18, 31, 33, 35, 39, 45, 51, 52, 56, 58, 59, 66, 68, 73, and 90. More details on the testing are given in [9, 21]. We further categorized genotypes by those included in the cobas^®^ HPV test (16, 18, 31, 33, 35, 39, 45, 51, 52, 56, 58, 59, 66, 68; Roche Diagnostics; Risch-Rotkreuz, Switzerland) used in clinical settings, those designated by the International Agency for Research on Cancer (IARC) as group 1 (carcinogenic: 16, 18, 31, 33, 35, 39, 45, 51, 52, 56, 58, 59) or group 2A (probably carcinogenic: 68) carcinogens [24], those included in the Gardasil (6, 11, 16, 18) and Gardasil 9 vaccines (6, 11, 16, 18, 31, 33, 45, 52, 58; Merck; Kenilworth, NJ), and those responsible for genital warts (6, 11). Participants whose samples contained insufficient DNA or otherwise resulted in inconclusive test results were denoted as invalid.

### Statistical analysis

Study data were collected and managed using REDCap electronic data capture tools hosted at the University of Michigan [25, 26]. Data were analyzed in R 3.6 [27], 2018–20.

We consider two main outcome variables: 1) presence of HPV in a participant’s oral sample at the baseline study visit and 2) presence of HPV in a participant’s first valid cervicogenital sample. We use baseline oral and first valid cervicogenital test because the cervicogenital substudy was rolled out after the main study began. We assessed sample prevalence of these two outcomes in their respective populations based on demographic and behavior variables derived from participant answers to the surveys. We also stratified the cohort into ages 18–22 years (college-age cohort) and ages 23+ (older cohort) for oral HPV prevalence. To estimate prevalence ratios, we ran univariable, log-binomial regression models for oral HPV prevalence predicted by the variables in Table 1 in the full cohort and for cervicogenital HPV prevalence in the substudy cohort. We also separately modeled oral HPV prevalence in the college-age and older cohorts. Participants with missing data for a predictor variable were excluded from that model. All statistics were evaluated at level of significance *α* = 0.05.

**Table 1:** Baseline characteristics of participants in the MHOC study (data collected in Ann Arbor, MI, 2015–17, analyzed 2018–2020).

	Full cohort (N = 394)		College-age cohort (N = 241)		Older adult cohort (N = 153)		Cervicogenital substudy cohort (N = 130)	
	%	n	%	n	%	n	%	n
**Age (years)**								
18	25%	99	41%	99	0%	0	27%	35
19–22	36%	142	59%	142	0%	0	33%	43
23–29	12%	49	0%	0	32%	49	11%	14
30–49	12%	46	0%	0	30%	46	14%	19
50+	15%	58	0%	0	38%	58	15%	19
**Sex**								
Female	67%	265	70%	168	63%	97	100%	130
Male	33%	129	30%	73	37%	56	0%	0
**Race**								
White	61%	240	54%	131	71%	109	62%	80
Asian	21%	83	31%	74	6%	9	20%	26
Black/Hispanic/multiracial/unknown	18%	71	15%	36	23%	35	18%	24
**Marital status**								
Never married	77%	302	98%	237	42%	65	75%	97
Married/partnered	19%	73	1%	2	46%	71	19%	25
Widowed/divorced/separated	4%	17	0%	1	10%	16	6%	8
**Sexual attraction**								
Mostly-to-only to the opposite sex	89%	41	87%	210	87%	133	92%	120
To both sexes equally or mostly-to-only to the same sex	10%	343	10%	25	10%	16	5%	6
**Circumcised (male only)**								
Yes	71%	91	66%	48	77%	43	---	---
No	29%	37	33%	24	23%	13	---	---
**Ever diagnosed with STI** *								
No	94%	369	98%	237	86%	132	93%	121
Yes	6%	25	2%	4	14%	21	7%	9
**HPV vaccination**								
No	44%	177	24%	59	77%	118	45%	58
Yes	49%	192	66%	158	22%	34	51%	66
**Alcohol use**								
Never or non-current	29%	115	31%	75	26%	40	28%	37
Current	69%	260	65%	157	74%	113	70%	91
**Ever cigarette use**								
Never	76%	300	87%	209	59%	91	79%	103
Ever	23%	91	12%	30	40%	61	20%	26
**Ever marijuana use**								
Never	52%	204	59%	141	41%	63	52%	68
Ever	43%	171	36%	87	55%	84	45%	59
**Has ever engaged in**								
Deep kissing								
Yes	84%	330	80%	194	89%	136	88%	115
No	16%	62	19%	46	10%	16	12%	15
Manual sex								
Yes	68%	268	60%	144	81%	124	73%	95
No	29%	116	38%	92	16%	24	25%	33
Vaginal, oral, or anal sex								
Yes	75%	296	63%	152	94%	144	78%	101
No	24%	95	36%	86	6%	9	22%	29
Vaginal sex								
Yes	64%	252	48%	116	89%	136	68%	89
No	35%	137	51%	122	10%	15	32%	41
Oral sex								
Yes	71%	279	60%	145	88%	134	75%	98
No	28%	110	39%	93	11%	17	25%	32
Anal sex								
Yes	22%	88	12%	30	38%	58	20%	26
No	76%	299	86%	208	60%	91	78%	102
Anilingus								
Yes	13%	50	7%	17	22%	33	10%	13
No	86%	340	92%	222	77%	118	90%	117
**Deep kissing partners**								
0 lifetime	16%	62	19%	46	10%	16	12%	15
0 past 6 months, >0 lifetime	13%	51	8%	20	20%	31	14%	18
1 past 6 months	42%	165	33%	80	56%	85	42%	54
2–4 past 6 months	20%	77	24%	59	12%	18	24%	31
5+ past 6 months	9%	37	15%	35	1%	2	9%	12
**Vaginal, oral, or anal sex partners**								
0 lifetime	24%	95	36%	86	6%	9	22%	29
0 past 6 months, >0 lifetime	12%	49	8%	19	20%	39	12%	16
1 past 6 months	43%	171	32%	77	61%	94	46%	60
2–4 past 6 months	15%	61	17%	42	12%	19	18%	23
5+ past 6 months	4%	15	6%	14	1%	1	2%	2
**Vaginal sex partners**								
0 lifetime	35%	137	51%	122	10%	15	32%	41
0 past 6 months, >0 lifetime	11%	45	4%	10	23%	35	12%	16
1 past 6 months	38%	151	26%	63	58%	88	40%	52
2–4 past 6 months	11%	45	14%	33	8%	12	15%	20
5+ past 6 months	3%	10	4%	9	1%	1	1%	1
**Oral sex (participant received) partners**								
0 lifetime	28%	110	39%	93	11%	17	25%	32
0 past 6 months, >0 lifetime	18%	72	10%	23	32%	49	18%	23
1 past 6 months	36%	140	31%	74	43%	66	40%	52
2–4 past 6 months	13%	51	13%	32	12%	19	15%	20
5+ past 6 months	4%	15	6%	15	0%	0	2%	3
**Oral sex (participant performed) partners**								
0 lifetime	28%	110	39%	93	11%	17	25%	32
0 past 6 months, >0 lifetime	23%	89	14%	34	36%	55	20%	26
1 past 6 months	35%	137	30%	72	42%	65	43%	56
2–4 past 6 months	19%	41	12%	28	8%	13	12%	16
5+ past 6 months	3%	11	4%	10	1%	1	0%	0
**Anal sex partners**								
0 lifetime	76%	299	86%	208	59%	91	78%	102
0 past 6 months, >0 lifetime	11%	45	2%	4	27%	41	10%	13
1 past 6 months	8%	32	8%	20	8%	12	9%	12
2–4 past 6 months	3%	11	2%	6	3%	5	1%	1
5+ past 6 months	0%	0	0%	0	0%	0	0%	0

Note: percentages may not add up to 100% as participants could refuse to answer questions.

*Other than HPV.

## Results

### Cohort demographics and potential risk factor prevalence

We recruited 394 participants, 241 in the college-age cohort (ages 18–22 years) and 153 in the older cohort (ages 23+); 130 participants from the full cohort were recruited to also participate in the cervicogenital substudy (Table 1). Approximately two-thirds of our sample was female, and approximately 60% of participants were white. The second most common race was Asian at approximately 20%, though most Asian participants were in the college-age cohort. The majority of participants had never been married, though this varied by age (p<0.001). Approximately 70% of male participants were circumcised. Of the 94% of participants who reported their HPV vaccination status, approximately half indicated that they were vaccinated; however, as expected, there were stark differences in vaccination by age, with 66% of the college-age cohort reporting vaccination compared to 22% of the older cohort (p<0.001). Current alcohol use was similar in the college-age (65%) and older cohort (74%), while a larger fraction of the older cohort reported ever smoking cigarettes (40% vs 12%; p<0.001) and ever using marijuana (55% vs 36%; p<0.001).

Participants also reported the number of partners in the last 6 months for a variety of intimate and sexual activities (Table 1). Approximately 70% of the full cohort reported at least one recent deep kissing partner, with similar percentages in the different age groups. Just over half (55%) of the full cohort reported at least one recent vaginal sex or oral sex partner, and only 22% reported ever engaging in anal sex. Fewer participants in the younger cohort reported recent vaginal, oral, or anal sex (56%) compared to the older cohort (74%; p<0.001). A comparison of cohort characteristic by HPV status is included in the S1 Table.

### HPV prevalence

We found that 10.0% of the 338 participants with a valid oral HPV test at baseline were positive for one or more of the genotypes tested (Table 2). Similarly, 20.0% of the 130 participants’ first valid cervicogenital HPV tests were positive for at least one genotype. When considering only genotypes in the cobas^®^ HPV test, currently the only test FDA-approved for clinical use (on physician-collected cervicogenital swabs), the prevalence of oral HPV was only 6.5%, while the prevalence of cervicogenital HPV was 10.8%. The distribution of genotypes in the oral and cervicogenital samples is given in the vaccination status and age-group (S2 Table).

**Table 2:** HPV prevalence in the oral cavity (valid tests at baseline) or cervicovaginal canal (at first valid test) in the MHOC study by genotype classification (data collected in Ann Arbor, MI, 2015–17, analyzed 2018–2020).

	Full cohort (N = 338)		College-age cohort (N = 241)		Older adult cohort (N = 153)		Cervicogenital substudy cohort (N = 130)	
	%	n	%	n	%	n	%	n
Any tested: 6, 11, 16, 18, 31, 35, 33 39, 45, 51, 52, 56, 58, 59, 66, 68, 73, 90	10.0%	34	12.6%	25	6.5%	9	20.0%	26
Clinical test high-risk: 16, 18, 31, 33, 35, 39, 45, 51, 52, 56, 58, 59, 66, 68	6.5%	22	8.0%	16	4.3%	6	10.8%	14
IARC Group 1 and 2A: 16, 18, 33, 31, 35, 39, 45, 51, 52, 56, 58, 59, 68	5.3%	18	7.0%	14	2.9%	4	4.6%	6
Gardasil 9 vaccine genotypes: 6, 11, 16, 18, 31, 33, 45, 52, 58	6.5%	22	8.5%	17	3.6%	5	3.8%	5
Gardasil vaccine genotypes: 6, 11, 16, 18	6.2%	21	8.0%	16	3.6%	5	1.5%	2
Genital warts genotypes: 6, 11	2.1%	7	2.5%	5	1.4%	2	1.5%	2

We found oral and cervicogenital HPV prevalence varied by demographic and behavioral variables, though almost no variable achieved significance in univariable log-binomial regression models (Table 3). Oral HPV prevalence did not differ by sex (10.5% in women vs 9.0% in men); this result held even after age-adjusting the sample. Consistent with the literature, both oral and genital HPV showed a pattern of higher prevalence in younger ages (*<*23) and older ages (50+), with lower prevalence in between. Oral but not cervicogenital HPV was higher in Asian participants, although this result is likely due to the fact that most Asian participants were in the younger cohort. Oral HPV prevalence was not statistically significantly higher in men who were uncircumcised (10.0% vs. 7.6%) and among those who reported ever being diagnosed with an STI (15.8% vs. 9.7%). There were no cervicogenital infections among women ever reporting an STI, but the sample size was small (*n* = 9).

**Table 3:** Prevalence of oral and cervicovaginal HPV and prevalence ratios (PR) by covariate in the MHOC study (data collected in Ann Arbor, MI, 2015–17, analyzed 2018–2020).

	Oral HPV prevalence (N = 338)				Oral HPV prevalence in the college-age cohort (N = 199)				Oral HPV prevalence in the older adult cohort (N = 139)				Cervicogenital HPV prevalence (N = 130)			
	%	PR	95% CI	N	%	PR	95% CI	N	%	PR	95% CI	N	%	PR	95% CI	N
**Age (years)**																
18	12.7%	1 (ref)		79	12.7%	1 (ref)		79	—	—	—	0	28.6%	1 (ref)		35
19–22	12.5%	0.99	(0.47, 2.09)	120	12.5%	0.99	(0.47, 2.09)	120	—	—	—	0	25.6%	0.90	(0.43, 1.86)	43
23–29	2.3%	0.18	(0.02, 1.36)	44	—	—	—	0	2.3%	1 (ref)		44	14.3%	0.50	(0.13, 2.00)	14
30–49	6.8%	0.54	(0.16, 1.85)	44	—	—	—	0	6.8%	3.00	(0.32, 27.7)	44	0.0%	0.00	—	19
50+	10.0%	0.77	(0.43, 1.74)	51	—	—	—	0	9.8%	4.31	(0.52, 35.5)	51	15.8%	0.55	(0.17, 1.77)	19
**Sex**																
Female	10.5%	1 (ref)		228	13.7%	1 (ref)		139	5.6%	1 (ref)		89	20.0%	1 (ref)		130
Male	9.0%	0.86	(0.39, 1.84)	110	10.0%	0.73	(0.31, 1.74)	60	8.0%	1.42	(0.40, 5.06)	50	—	—	—	0
**Race**																
White	9.6%	1 (ref)		207	12.0%	1 (ref)		108	7.1%	1 (ref)		99	20.0%	1 (ref)		80
Asian	15.7%	1.63	(0.82, 3.22)	70	16.4%	1.36	(0.64, 2.92)	61	11.1%	1.57	(0.22, 11.4)	9	19.2%	0.96	(0.31, 2.37)	26
Black/Hispanic/multiracial/unknown	9.1%	0.94	(0.24, 3.76)	22	18.1%	1.51	(0.39, 5.85)	11	0.0%	—	—	11	0.0%	0.00	—	10
**Marital status**																
Never married	10.6%	1 (ref)		255	12.2%	1 (ref)		196	5.1%	1 (ref)		59	22.7%	1 (ref)		97
Married/partnered	6.2%	0.58	(0.21, 1.60)	65	††	††	††	1	6.3%	1.23	(0.29, 5.26)	64	16.0%	0.71	(0.27, 1.86)	25
Widowed/divorced/separated	12.5%	1.18	(0.31, 4.53)	16	††	††	††	1	13.3%	2.62	(0.48, 14.3)	15	0.0%	0.00	—	8
**Sexual attraction**																
Mostly-to-only to opposite sex	11.0%	1 (ref)		292	14.2%	1 (ref)		169	6.5%	1 (ref)		123	20.0%	1 (ref)		120
To both sexes equally or mostly-to-only to same sex	2.7%	0.25	(0.03, 1.75)	37	0.0%	—	—	24	7.7%	1.18	(0.16, 8.73)	13	16.7%	0.83	(0.13, 5.17)	6
**Circumcised (male only)**																
Yes	7.6%	1 (ref)		79	7.5%	1 (ref)		40	7.7%	1 (ref)		39	—	—	—	—
No	10.0%	1.32	(0.35, 4.93)	30	10.5%	1.40	(0.26, 7.71)	19	9.1%	1.18	(0.14, 10.3)	11	—	—	—	—
**Ever diagnoses with STI** *																
No	9.7%	1 (ref)		319	12.8%	1 (ref)		196	4.9%	1 (ref)		123	21.5%	1 (ref)		121
Yes	15.8%	1.62	(0.55, 4.84)	19	††	††	††	3	18.8%	3.84	(1.06, 13.9)	16	0.0%	0.00	—	9
**HPV vaccination**																
No	9.4%	1 (ref)		159	14.0%	1 (ref)		50	7.34%	1 (ref)		109	18.9%	1 (ref)		58
Yes	10.1%	1.07	(0.55, 2.08)	159	11.6%	0.83	(0.36, 1.92)	129	3.33%	0.45	(0.06, 3.49)	30	22.7%	1.20	(0.60, 2.40)	66
**Alcohol use**																
Never or non-current	10.4%	1 (ref)		96	14.8%	1 (ref)		61	2.9%	1 (ref)		35	21.6%	1 (ref)		91
Current	9.4%	0.90	(0.44, 1.83)	234	10.8%	0.73	(0.33, 1.59)	130	7.7%	2.69	(0.35, 20.8)	104	18.7%	0.86	(0.41, 1.83)	37
**Ever cigarette use**																
Never	10.9%	1 (ref)		257	13.1%	1 (ref)		175	6.1%	1 (ref)		82	0.22	1 (ref)		103
Ever	7.5%	0.69	(0.30, 1.60)	80	8.3%	0.63	(0.16, 2.52)	24	7.1%	1.17	(0.33, 4.17)	56	0.12	0.52	(0.17, 1.59)	26
**Ever marijuana use**																
Never	9.8%	1 (ref)		150	13.9%	1 (ref)		115	1.7%	1 (ref)		75	22.1%	1 (ref)		68
Ever	8.7%	0.88	(0.44, 1.76)	173	9.3%	0.67	(0.29, 1.55)	75	8.0%	4.64	(0.57, 37.5)	58	18.6%	0.84	(0.42, 1.69)	59
**Has ever engaged in** **																
Deep kissing	8.2%	**0.40**	**(0.21, 0.77)**	282	10.1%	0.44	(0.21, 0.91)	159	5.7%	0.43	(0.10, 1.87)	123	18.3%	0.54	(0.24, 1.23)	115
Manual sex	9.0%	0.72	(0.37, 1.42)	233	10.8%	0.74	(0.35, 1.56)	120	7.1%	1.56	(0.20, 11.8)	113	20.0%	0.94	(0.44, 2.04)	95
Vaginal, oral, or anal sex	8.9%	0.69	(0.35, 1.07)	257	11.1%	0.78	(0.36, 1.66)	126	6.9%	—	—	131	18.9%	0.78	(0.36, 1.67)	101
Vaginal sex	8.5%	0.68	(0.35, 1.30)	223	10.2%	0.71	(0.33, 1.53)	98	7.2%	—	—	125	18.0%	0.74	(0.37, 1.48)	89
Oral sex	8.6%	0.65	(0.33, 1.28)	243	9.9%	0.62	(0.29, 1.31)	121	7.4%	—	—	122	19.4%	0.88	(0.41, 1.91)	98
Anal sex	9.0%	0.88	(0.40, 1.94)	254	12.0%	0.97	(0.31, 3.03)	25	7.5%	1.25	(0.35, 4.46)	53	15.4%	0.71	(0.27, 1.89)	26
Anilingus	6.8%	0.64	(0.20, 2.00)	44	7.1%	0.55	(0.08, 3.75)	14	6.7%	1.02	(0.22, 4.65)	30	15.4%	0.75	(0.20, 2.81)	13
**Deep kissing partners**																
0 lifetime	20.3%	2.19	(0.75, 6.40)	54	23.1%	3.92	(0.54, 28.6)	39	13.3%	1.16	(0.22, 6.15)	15	33.3%	3.00	(0.68, 13.3)	15
0 past 6 months, >0 lifetime	9.3%	1 (ref)		43	5.9%	1 (ref)		17	11.5%	1 (ref)		26	11.1%	1 (ref)		18
1 past 6 months	7.8%	0.84	(0.28, 2.50)	141	13.1%	2.23	(0.30, 16.6)	61	3.8%	0.33	(0.07, 1.51)	80	9.3%	0.83	(0.18, 3.93)	54
2–4 past 6 months	6.3%	0.68	(0.18, 2.58)	63	8.3%	1.42	(0.17, 11.8)	48	0.0%	—	—	15	29.0%	2.6	(0.63, 10.8)	31
5+ past 6 months	11.4%	1.23	(0.33, 4.56)	35	9.1%	1.54	(0.17, 13.8)	33	††	††	††	2	41.7%	3.75	(0.86, 16.3)	12
**Vaginal, oral, or anal sex partners**																
0 lifetime	12.8%	0.75	(0.31, 1.83)	78	14.3%	0.67	(0.21, 2.12)	70	0.0%	0.00	—	8	24.1%	—	—	29
0 past 6 months, >0 lifetime	17.1%	1 (ref)		41	21.4%	1 (ref)		14	14.8%	1 (ref)		27	0.0%	1 (ref)		16
1 past 6 months	6.7%	**0.39**	**(0.16, 0.96)**	150	9.5%	0.44	(0.13, 1.57)	63	4.6%	0.31	(0.08, 1.16)	87	15.0%	—	—	60
2–4 past 6 months	7.5%	0.44	(0.14, 1.41)	53	8.1%	0.38	(0.09, 1.66)	37	6.3%	0.42	(0.05, 3.45)	16	39.1%	—	—	23
5+ past 6 months	15.4%	0.90	(0.21, 3.81)	13	16.7%	0.78	(0.15, 3.91)	14	—	—	—	0	††	—	—	2
**Vaginal sex partners**																
0 lifetime	12.6%	0.78	(0.32, 1.88)	111	14.3%	0.43	(0.13, 1.47)	98	0.0%	0.00	—	13	24.3%	—	—	41
0 past 6 months, >0 lifetime	16.2%	1 (ref)		37	33.3%	1 (ref)		6	12.9%	1 (ref)		31	0.0%	1 (ref)		16
1 past 6 months	5.1%	**0.31**	**(0.11, 0.87)**	138	7.3%	**0.22**	**(0.05, 0.95)**	55	3.6%	0.28	(0.07, 1.18)	83	13.5%	—	—	52
2–4 past 6 months	7.9%	0.48	(0.13, 1.80)	38	7.1%	0.21	(0.04, 1.23)	28	10.0%	0.78	(0.10, 6.15)	10	45.0%	—	—	20
5+ past 6 months	33.3%	2.06	(0.63, 6.68)	9	25.0%	0.75	(0.14, 3.90)	8	†	†	†	1	††	—	—	1
**Oral sex (participant received) partners**																
0 lifetime	13.2%	1.05	(0.46, 2.43)	91	16.0%	1.07	(0.33, 3.41)	75	0.0%	0.00	—	16	21.9%	2.21	(0.57, 11.0)	32
0 past 6 months, >0 lifetime	12.5%	1 (ref)		64	15.0%	1 (ref)		20	11.4%	1 (ref)		44	8.7%	1 (ref)		23
1 past 6 months	5.9%	0.47	(0.18, 1.25)	118	8.6%	0.57	(0.15, 2.19)	58	3.3%	0.29	(0.06, 1.44)	60	19.2%	2.21	(0.53, 9.30)	52
2–4 past 6 months	10.6%	0.85	(0.30, 2.44)	47	10.3%	0.69	(0.15, 3.08)	29	11.1%	0.98	(0.21, 4.59)	18	30.0%	3.45	(0.78, 15.2)	20
5+ past 6 months	7.7%	0.62	(0.08, 4.51)	13	7.7%	0.51	(0.06, 4.41)	13	—	—	—	0	††	††	††	3
**Oral sex (participant performed) partners**																
0 lifetime	13.1%	1.02	(0.46, 2.22)	91	16.0%	0.93	(0.35, 2.40)	75	0.0%	0.00	—	16	21.9%	2.84	(0.64, 12.5)	32
0 past 6 months, >0 lifetime	13.0%	1 (ref)		77	17.2%	1 (ref)		29	10.4%	1 (ref)		48	7.7%	1 (ref)		26
1 past 6 months	5.0%	0.38	(0.14, 1.01)	121	6.9%	0.40	(0.12, 1.37)	58	3.2%	0.30	(0.06, 1.50)	63	17.9%	2.32	(0.54, 9.85)	56
2–4 past 6 months	14.3%	1.10	(0.40, 2.98)	35	12.0%	0.70	(0.18, 2.63)	25	20.0%	1.92	(0.43, 8.53)	10	43.8%	**5.69**	**(1.34, 24.1)**	16
5+ past 6 months	0.0%	0.00	—	9	0.0%	0.00	—	8	††	††	††	1	—	—	—	0
**Anal sex partners**																
0 lifetime	10.2%	1.36	(0.43, 4.30)	254	12.2%	††	††	171	6.0%	1.14	(0.23, 5.64)	83	21.2%	2.80	(0.41, 19.1)	102
0 past 6 months, >0 lifetime	7.5%	1 (ref)		40	††	1 (ref)	††	2	5.3%	1 (ref)		38	7.7%	1 (ref)		13
1 past 6 months	7.4%	0.98	(0.18, 5.52)	27	5.9%	††	††	17	10.0%	1.90	(0.19, 18.9)	10	25.0%	3.25	(0.39, 27.2)	12
2–4 past 6 months	18.2%	2.42	(0.46, 12.8)	11	16.7%	††	††	6	20.0%	3.80	(0.42, 34.1)	5	††	††	††	1

PRs significant at level α = 0.05 are indicated in bold.

*Other than HPV.

**Prevalence ratios are vs “has never engaged in.”

†Number of partners in the past 6 months among those who ever engaged in the behavior.

††Cells with fewer than 5 participants are censored.

There was little difference in oral or cervicogenital prevalence among those who reported being vaccinated and those who reported being unvaccinated, even when considering only genotypes 6, 11, 16, and 18 (oral prevalence among vaccinated (6.3%) and unvaccinated (6.3%); cervicogenital prevalence among vaccinated (1.5%) and unvaccinated (1.7%)) although the absolute numbers of positives are small. The apparent lack of protection from vaccination appears to be the result of an ecological fallacy, however. When considering the college-age and older adult cohort separately, the point estimates of oral HPV prevalence were lower in vaccinated individuals of both groups.

Those reporting ever engaging in deep kissing (0.4, 95% CI: 0.21, 0.77) or any sexual behavior (not statistically significant) had lower oral and cervicogenital HPV prevalence than those that did not (Table 3). This result is driven by the high oral and cervicogenital HPV prevalence among those who reported no sexual behavior in the college-age cohort. In contrast, prevalence among those reporting no sexual behavior in the older adult (age 23+) cohort was consistently low or 0. When examining the number of recent sexual partners of each type, we generally found that reporting one recent (past 6 months) partner was associated with lower oral HPV prevalence than reporting no recent (but at least one ever) partners of each type; this result achieved significance for any sex (PR 0.39, 95% CI: 0.16, 0.96) and for vaginal sex (PR 0.31, 95% CI: 0.11, 0.87) in the full cohort and was in the correct direction but non-significant for oral sex. Point estimates of oral HPV prevalence for those reporting more than 1 recent sexual partner were generally higher than those reporting only 1 but did not achieve significance because of low power. For cervicogenital HPV prevalence, on the other hand, we did not find higher prevalence among those reporting no sexual partners of each type. Indeed, cervicogenital HPV prevalence among those reporting no recent vaginal sex partners was 0.0% (n = 16), and point estimates of cervicogenital HPV prevalence increased in a gradient with more partners of each type, although this result only achieved significance for 2–4 recent partners on whom the participant performed oral sex (PR 5.69, 95% CI: 1.34, 24.1).

## Discussion

This analysis presents a first look at the Michigan HPV and Oropharyngeal Cancer (MHOC) Study’s epidemiological cohort. In this baseline analysis, we investigated associations of HPV prevalence with demographic and behavioral risk factors. Compared to the nationally representative National Health and Nutrition Examination Survey [10, 11], we find much higher oral HPV prevalence among women (NHANES 2009–14: 3.4%; MHOC: 10.5%), especially considering that NHANES tests for 37 genotypes while MHOC tested for 18. For IARC Group 1 HPV types [24], oral prevalence in women in NHANES 2009–14 was 1.3%, compared to 6.0% in MHOC. For men, Group 1 oral HPV prevalence in NHANES was 5.6% (11.1% for any tested genotype) and in MHOC was 4.8% (9.0% for any tested). Interestingly, the high oral HPV prevalence in women is not matched by a high cervicogenital prevalence; Group 1 cervicogenital prevalence in NHANES was 18.6% (40.6% for any tested genotype) and in MHOC was 4.6% (20.0% for any tested). In MHOC, oral HPV prevalence in women was higher than men in the younger cohort (13.7% vs. 10.0%) but lower in the older cohort (5.6% vs 8.0%). Although the MHOC study was not designed to be nationally representative, these differences persist after age-adjusting the prevalence. Given the similarity in prevalence between NHANES and MHOC among men, the differences between the two studies are unlikely to be purely based on assay sensitivity or laboratory methods. One explanation for the high HPV prevalence in women in MHOC is that prevalence of HPV—both overall prevalence and genotype-specific prevalence—may vary regionally. It is also possible that the risk behaviors of MHOC participants differ from the national average in ways that make oral infection more likely and cervicogenital HPV less likely.

However, while the prevalence magnitudes differ between NHANES and MHOC, the age-specific patterns are very similar, both exhibiting the classic bimodal distribution [28]: high prevalence among the youngest and oldest age groups, with lower prevalence in between. This underscores the importance of examining age and cohort patterns in HPV prevalence, as the increase in higher age groups may be the result of generational (birth-cohort) differences, increased sexual activity at older ages, or reactivation of HPV at older ages [10, 29–31].

We found high oral and cervicogenital prevalence among college-age participants who reported no sexual partners in their lifetime. It may that these participants were not reporting their experiences accurately, possibly to avoid consequences if their answers were ever disclosed or simply to minimize the survey burden. In any case, we believe that our analysis of the associations of sexual behavior and HPV prevalence are subject to some level of misclassification bias. We also found that oral HPV prevalence was generally higher among those reporting at least one lifetime but no recent partners compared to those reporting one recent partner, and this result was consistent for both age groups. However, we did not find an analogous result for cervicogenital HPV; those reporting no recent sexual partners had lower than those reporting at least one. When considering only those who report at least one recent sexual partner, prevalence increased with the number of recent sex partners, as expected. While sexual acts are strongly correlated, previous studies have differed in the reported strength of association between oral vs. vaginal sex and oral HPV prevalence [12–15]; here, we find that both oral and cervicogenital HPV prevalence are strongly associated with number of recent vaginal sex partners (among those reporting ever having had vaginal sex). The number of oral sex partners (among those reporting ever having had oral sex), while still positively associated with both oral and cervicogenital HPV prevalence, was not substantially stronger than the number of recent deep kissing partners (among those reporting ever deep kissing). Although there is reason to believe that risk differs based on the sex of one’s partner [16], we were not powered to investigate these differences in this study.

Nationally representative data demonstrate that oral and genital prevalence of HPV vaccine genotypes is reduced among vaccinated people [10, 18]. In this analysis, we were not able to find strong evidence of a reduction in prevalence likely due to not having a large enough sample size. In addition, the lack of difference at the cohort level is, in part, an ecological fallacy, as the point estimates of the effect of vaccination on infection prevalence were in the protective direction when considering the younger and older cohorts separately. Misreporting of vaccination status likely accounts for the smaller than expected observed effect of vaccination in this analysis. Vaccination is also known to be less effective in people already exposed to HPV [32], which may further attenuate effects here, particularly in the older cohort.

The limitations of this baseline analysis include the relatively small sample size compared to NHANES and other HPV prevalence studies. However, because this cohort is designed to be studied longitudinally, we will have substantially more power in the analysis of HPV prevalence over time. Analysis of the longitudinal data will later provide a detailed consideration of the short-term dynamics of the detection of oral and cervicogenital HPV [33]. This in turn will aid in the development of oral HPV and sexual behavior transmission models, and models of the natural history of oropharyngeal cancer [21, 34]. Other limitations of the study are the self-reported nature of sexual and other behaviors and vaccination, which are subject to misclassification.

This hypothesis-generating work contributes to our understanding of the prevalence and determinants of oral HPV by suggesting that prevalence of HPV in women is not uniformly lower than in men. More work is needed to understand regional variation in oral HPV prevalence overall and by genotype. Too, more work is needed to better understand the specific transmission pathways that lead to oral HPV infection, particularly the relative contribution of oral sex vs autoinoculation from genital infection and the role of latent infections.

## Supporting information

S1 Table Baseline characteristics of participants in the MHOC study by oral HPV status. Data collected in Ann Arbor, MI, 2015–17, analyzed 2018–2020. Note: percentages may not add up to 100% as participants could refuse to answer questions. *Other than HPV.
(DOCX)

S2 Table Oral and cervicogenital HPV genotypes by age group and vaccination status. (DOCX)

S1 File Minimal, deidentified data. This minimal, deidentified data set contains the baseline demographic, behavioral, and HPV data on each MHOC Study participant.
(CSV)
