# Supplementary material for: Prevalence and determinants of oral and cervicogenital HPV infection: Baseline analysis of the Michigan HPV and Oropharyngeal Cancer (MHOC) cohort study
Source: PLoS One. 2022 May 16;17(5):e0268104. doi: 10.1371/journal.pone.0268104 (PMC9109914; doi:10.1371/journal.pone.0268104)
Supplement: S2 Table — (DOCX) [file pone.0268104.s002.docx]

**S2 Table: Oral and cervicogenital HPV genotypes by age group and vaccination status.**

|  | Oral HPV  (N=338) | | | | | | | | | | Cervical HPV  (N=130) | | | | | |
| --- | --- | --- | --- | --- | --- | --- | --- | --- | --- | --- | --- | --- | --- | --- | --- | --- |
|  | All  (N=338) | | College-age cohort  (N=241) | | Older cohort  (N=153) | | Vaccinated  (N=192) | | Unvaccinated  (N=177) | | All  (N=130) | | Vaccinated  (N=66) | | Unvaccinated  (N=58) | |
|  | % HPV+ | n | % HPV+ | n | % HPV+ | n | % HPV+ | n | % HPV+ | n | % HPV+ | N | % HPV+ | N | % HPV+ | N |
| HPV 6 | 2% | 7 | 2% | 5 | 1% | 2 | 1% | 2 | 3% | 5 | 2% | 2 | 2% | 1 | 2% | 1 |
| HPV 11 | 1% | 2 | 1% | 2 | 0% | 0 | 0% | 0 | 1% | 2 | 0% | 0 | 0% | 0 | 0% | 0 |
| HPV 16 | 2% | 6 | 2% | 6 | 0% | 0 | 2% | 4 | 1% | 1 | 2% | 2 | 2% | 1 | 2% | 1 |
| HPV 18 | 2% | 8 | 2% | 5 | 2% | 3 | 2% | 4 | 2% | 4 | 1% | 1 | 0% | 0 | 2% | 1 |
| HPV 31 | 0% | 0 | 0% | 0 | 0% | 0 | 0% | 0 | 0% | 0 | 1% | 1 | 0% | 0 | 2% | 1 |
| HPV 33 | 0% | 0 | 0% | 0 | 0% | 0 | 0% | 0 | 0% | 0 | 0% | 0 | 0% | 0 | 0% | 0 |
| HPV 35 | 0% | 1 | 0% | 0 | 1% | 1 | 1% | 1 | 0% | 0 | 0% | 0 | 0% | 0 | 0% | 0 |
| HPV 39 | 0% | 0 | 0% | 0 | 0% | 0 | 0% | 0 | 0% | 0 | 1% | 1 | 2% | 1 | 0% | 0 |
| HPV 45 | 0% | 0 | 0% | 0 | 0% | 0 | 0% | 0 | 0% | 0 | 1% | 1 | 2% | 1 | 0% | 0 |
| HPV 51 | 0% | 0 | 0% | 0 | 0% | 0 | 0% | 0 | 0% | 0 | 2% | 2 | 2% | 1 | 2% | 1 |
| HPV 52 | 0% | 1 | 0% | 1 | 0% | 0 | 0% | 0 | 0% | 0 | 5% | 6 | 5% | 3 | 5% | 3 |
| HPV 56 | 1% | 2 | 1% | 2 | 0% | 0 | 1% | 2 | 0% | 0 | 2% | 2 | 0% | 0 | 3% | 2 |
| HPV 58 | 0% | 1 | 0% | 1 | 0% | 0 | 0% | 0 | 0% | 0 | 1% | 1 | 0% | 0 | 2% | 1 |
| HPV 59 | 0% | 0 | 0% | 0 | 0% | 0 | 0% | 0 | 0% | 0 | 5% | 6 | 6% | 4 | 3% | 2 |
| HPV 66 | 1% | 4 | 1% | 2 | 1% | 2 | 0% | 0 | 2% | 4 | 8% | 11 | 8% | 5 | 9% | 6 |
| HPV 68 | 0% | 0 | 0% | 0 | 0% | 0 | 0% | 0 | 0% | 0 | 0% | 0 | 0% | 0 | 0% | 0 |
| HPV 73 | 2% | 2 | 0% | 1 | 1% | 1 | 0% | 0 | 1% | 2 | 2% | 3 | 3% | 2 | 2% | 1 |
| HPV 90 | 2% | 7 | 2% | 7 | 0% | 0 | 3% | 5 | 1% | 1 | 7% | 9 | 6% | 4 | 10% | 5 |
